# Refugees

**DOI:** 10.4269/ajtmh.24-0728

**Published:** 2024-11-05

**Authors:** Philip J. Rosenthal

**Affiliations:** Department of Medicine, University of California, San Francisco, California

I was impressed by how unimpressive the scene appeared. We were passing through a rural African village. Roadside shops offered food and beverages, basic goods, mobile phone minutes – the usual offerings anywhere. Nearby homes were simple, but well-kept. The planted fields were well tended. Routine activities appeared to go on without particular fuss. Yet, this was not a typical African village, but rather a large refugee camp a few kilometers away from a major war zone in South Sudan. With little drama, families escaping the horrors of war were provided care, shelter, and the opportunity to restart their lives. As we hear frequently of overwhelming refugee challenges worldwide, it was uplifting to see a system that, by all accounts, successfully, albeit quietly, manages large numbers of refugees under challenging circumstances.

In preparation for studying malaria in newly arrived and settled refugees, I visited refugee settlement centers in northern Uganda with members of our Ugandan and American research team. As a translational medical researcher, I’m not very qualified to judge the quality of services for refugees. I was on this trip to explore the best means of studying malaria, not to gauge the success of refugee care. But, I could not help noticing that, in this region beset by conflict, a system for addressing the refugee crisis appeared to be working – indeed working better than systems developed in the much wealthier Global North.

At Elegu, on the South Sudanese border, the new arrivals lugging their sparse belongings just a few meters from the border looked more anxious than relieved. Here, refugees fleeing violence and food insecurity are registered, evaluated for potentially dangerous communicable conditions such as cholera, Ebola, or hemorrhagic fever virus infections, and treated for active infections such as malaria. Hundreds arrive each month.

A few kilometers away was the more comprehensive reception center, where the refugees are next sent, on or soon after the day of arrival. Here, in a large field filled with dozens of simple buildings designed to provide services and shelter, refugees are housed for weeks to months until long-term placement can be finalized. The refugees receive housing, food, and additional evaluation and medical care as indicated. At the reception center, there was calm. The children were playful, and the adults seemed to be relaxed, aware that they were now safe, but we saw the effects of what they had been through: set fractures in adults, and the flaxen hair and swollen bellies of malnutrition in some of the children. We could only guess what stories these recent arrivals could tell.

About an hour’s drive away, including a ferry across the Nile, is Palorinya, where ∼130,000 refugees have been placed in a rural district. Here, refugees begin their lives anew, with the opportunity to build simple housing and farm small plots. Their children attend local schools, allowing integration with local communities. Nearby shops, run by both refugees and members of the local population, offer basic services. The refugee camp might be mistaken for any rural African village, except that it is bigger and probably more organized than most, and that it includes nearby administrative complexes and warehouses managed by the government of Uganda and non-governmental organizations. With the exception of these additional facilities, the camp blends into the local community, and it was difficult for me to appreciate which homes and shops were in the refugee camp and which were part of the local community.

We focused on health care. Facilities at the short-term reception center are limited, with the ability to diagnose malaria and consider worrisome epidemic conditions. At the stable settlement, in contrast, care is integrated with the community, with health centers serving both refugee and native populations. Remarkably, care at the settlement may be superior to that of many rural African health centers, benefitting from investment both from the Uganda Ministry of Health and from multiple non-governmental organizations. The health center that we visited boasted more staff at all levels of training than typical for a rural center. Testing for numerous infectious or non-infectious conditions was available; some tests are completed at the local health center, and some are sent out to regional hospitals. Services are basic (e.g. receipt of routine chemistry results requires about a one-week turnaround), but attuned to local needs, with, it seemed based on discussion with the director, fewer problems with stockouts of medicines or supplies than often seen at rural African health centers. Ironically, after experiencing the dangers of fleeing a war zone, settled refugees may experience a more stable healthcare setting than that available to many Africans.

Still, life is not easy for refugees in Uganda. Returning home to their countries of origin is generally not an option for families. We heard that men often return to fight in ongoing conflicts. At the settlements, reports from the United Nations High Commissioner for Refugees and others indicate that cuts in outside assistance have led to decreased aid, compromising available services. Climate change–associated weather events and unpredictable markets challenge stable housing and agricultural activities. Refugees are free to travel in Uganda, but they must remain in settlements to receive aid. Overall, refugees benefit from a reasonably supportive living situation, but opportunities for long-term stability and economic advancement are limited.

Why do I tell this undramatic story? It is not to catalogue the myriad of human miseries that lead to refugee crises around the world. These stories are recounted again and again, and they should be, to remind us of the travails of those whom, due to chance, suffer great misfortune. But, I leave that telling to others. Rather, my interest is to comment, at a time of growing anti-immigrant sentiment in the Global North, on how refugee challenges can be managed with reasonable success. In the North, politicians bloviate about influxes of refugees, implying that the unfortunate victims are the problem. We need to put up walls, they argue. But, to my superficial inspection, these refugees were not rapists or escapees from mental hospitals, as claimed by one prominent politician, but rather normal families trying to survive under difficult circumstances. One assumes that the drive and determination that led them to travel long distances to safety will translate into productive activities in their new homes. And it was remarkable to see Uganda respond to the problem not by putting up walls, but by building supportive settlements, integrated into the national framework.

I appreciate that the welcoming of refugees may not arise only out of altruism. Care for refugees is likely a good business proposition for many in host communities. Indeed, studies have shown economic benefits for regions hosting refugees. Nonetheless, it was refreshing to see refugees welcomed and treated with dignity. We can hope that the wealthiest countries of the world, which are most equipped to help, but lately most inclined to attack the unfortunate refugees as the problem and to close their borders, can learn from Uganda about practical, compassionate care of the unfortunate victims of conflict.

